# Editorial: Recent Insights Into the Double Role of Hydrogen Peroxide in Plants

**DOI:** 10.3389/fpls.2022.843274

**Published:** 2022-01-28

**Authors:** Naser A. Anjum, Sarvajeet Singh Gill, Francisco J. Corpas, Cristina Ortega-Villasante, Luis E. Hernandez, Narendra Tuteja, Adriano Sofo, Mirza Hasanuzzaman, Masayuki Fujita

**Affiliations:** ^1^Department of Botany, Aligarh Muslim University, Aligarh, India; ^2^Stress Physiology and Molecular Biology Lab, Centre for Biotechnology, MD University, Rohtak, India; ^3^Department of Biochemistry, Cell and Molecular Biology of Plants, Experimental Station of Zaidín, Spanish National Research Council (CSIC), Granada, Spain; ^4^Fisiología Vegetal (Plant Physiology Laboratory), Dpto. Biología (Biology Department), Universidad Autónoma de Madrid, Madrid, Spain; ^5^Laboratory of Plant Physiology, Department of Biology/Research Centre for Biodiversity and Global Change, Universidad Autónoma Madrid, Madrid, Spain; ^6^Plant Molecular Biology Group, International Centre for Genetic Engineering and Biotechnology (ICGEB), New Delhi, India; ^7^Department of European and Mediterranean Cultures: Architecture, Environment, and Cultural Heritage (DICEM), University of Basilicata, Potenza, Italy; ^8^Department of Agronomy, Faculty of Agriculture, Sher-e-Bangla Agricultural University, Dhaka, Bangladesh; ^9^Laboratory of Plant Stress Responses, Department of Applied Biological Science, Faculty of Agriculture, Kagawa University, Takamatsu, Japan

**Keywords:** abiotic stresses, hydrogen peroxide, H_2_O_2_-metabolism, priming, plant stress tolerance

Reactive oxygen species (ROS) of varied types can be yielded in plants at several primary sites (such as the chloroplast, mitochondria, and peroxisomes) under normal aerobic metabolism *via* processes including photosynthetic and respiratory electron transport chains. However, impaired oxidant-antioxidant balance and extreme growth conditions in plants are bound to cause increases in the cellular concentrations of radical and non-radical ROS such as superoxide anions (O^2•−^), hydroxyl radical (OH•), singlet oxygen (_1_O^2^), and hydrogen peroxide (H_2_O_2_). On the one hand, H_2_O_2_ has no unpaired electrons and is moderately reactive. Owing to its relative stability compared to other ROS and its capacity for diffusing through aquaporins in the membranes and over more considerable distances within the cell (Bienert et al., 2007), H_2_O_2_ acts as a stress signal transducer and contributes to numerous physiological functions in plants. On the other hand, H_2_O_2_ is a relatively long-lived molecule with a half-life of 1 ms, readily crosses biological membranes, and consequently can bring oxidative consequences far from the site of its formation (Neill et al., 2002; Sharma et al., 2012; Sehar et al., 2021). The Frontiers Research Topic “*Recent Insights into the Double Role of Hydrogen Peroxide in Plants*” highlighted the major mechanisms underlying the dual role of H_2_O_2_ in response to different abiotic stresses in plants. This Research Topic incorporated 19 publications, including 10 original research articles, 8 reviews, and one perspective article.

## H_2_O_2_-Metabolism and H_2_O_2_-Priming Roles in Abiotic Stress Management

As a potent signaling molecule H_2_O_2_ gets produced in routine in stressed or non-stressed conditions *via* dismutation of O^2•−^ radicals through superoxide dismutase (SOD) during electron transport in different compartments of the plant cell, and is involved in the regulation of the plant growth, metabolism, and stress tolerance. It has also been noted that at higher concentrations in the cell during oxidative stress, ROS, including H_2_O_2_, can oxidize vital biomolecules (like nucleic acids, proteins, and lipids) and significantly impacts the seed germination process (Wojtyla et al.). Among the major abiotic stress factors, several heavy metals provoke increases in the production of ROS through plasma membrane-bound NADPH oxidases. However, the relationship of H_2_O_2_ has also been established in heavy metal tolerance in crop plants (Cuypers et al.). H_2_O_2_ directly mediates metal-induced oxidative signaling, where the production of H_2_O_2_ may involve H_2_O_2_ receptors, redox-sensitive transcription factors and inhibition of phosphatases (Miller et al., 2008). H_2_O_2_ sensing in metal-exposed plants also involves activation of mitogen-activated protein kinase (MAPK) pathways (Opdenakker et al., 2012). Additionally, interaction of H_2_O_2_ with Ca^2+^ (Baliardini et al., 2015), NO (Arasimowicz-Jelonek et al., 2012) and oxylipins (Tamás et al., 2009; Keunen et al., 2013) was also reported in metal-exposed plants. Though excess accumulation of H_2_O_2_ and polyamines (PAs) can be detrimental for the plant cell leading to premature cell death, a fine-tuning of these signaling molecules (H_2_O_2_ and PAs) can result in stress management by coordinating intra-cellular and systemic signaling systems (Gupta et al.). Polyamine oxidase (PAO)-induced production of H_2_O_2_ was found to be involved in the coleorhiza-limited rice seed germination (Chen et al.). ROS-specific probe DCFH2-DA enabled confocal laser scanning microscopy revealed a high level of ROS in the stigma at different developmental stages (unopened flower buds, recently opened flowers, dehiscent anthers, and flowers after fertilization) of scrutinized plants (Zafra et al.).

During evolution, plants have developed an efficient ROS-scavenging system constituting an array of enzymatic (SOD; CAT, catalase; APX, ascorbate peroxidase; GR, glutathione reductase; MDHAR, monodehydroascorbate reductase; DHAR, dehydroascorbate reductase; GPX, glutathione peroxidase; GOPX, guaiacol peroxidase, and GST, glutathione-S-transferase) and non-enzymatic (AsA, ascorbic acid; GSH, glutathione; phenolic compounds, alkaloids, non-protein amino acids, and α-tocopherols) antioxidants to get rid of excessive ROS in the cell (Singh et al.). Notably, NADH oxidase (RBOH), alternative oxidase (AOX), the plastid terminal oxidase (PTOX), and the malate valve with the malate dehydrogenase isoforms are involved in maintenance of the cellular redox homeostasis under salinity stress (Hossain and Dietz). In *Arabidopsis* cell suspension cultures, anoxia stress/shock led to significant increases in H_2_O_2_ (and also nitric oxide, NO); however, re-oxygenation maintained the components of ROS scavenging machinery like ascorbate-glutathione (AsA-GSH) system, α-tocopherol, and eventual cell survival as result of decreased H_2_O_2_ (Paradiso et al.). *Eutrema salsugineum* (halophyte) and *Arabidopsis thaliana* (glycophyte) exhibited a differential pattern of accumulation and scavenging of ROS. In particular, compared to *A. thaliana* chloroplasts, *E*. *salsugineum* chloroplasts showed a constitutive increase and the cell's steady-state regulation of H_2_O_2_ level which prepared this plant for ROS-control mainly due to an efficient ROS-scavenging machinery including glucosinolates content and well-coordinated tuning of hormonal signaling (Pilarska et al.). Elevation in the cellular level of H_2_O_2_ and its consequences can be controlled by brassinosteroids, a class of plant-specific essential steroid hormones. To this end, in tomato seedlings, brassinosteroid (24-epibrassinolide) ameliorated the impacts of zinc oxide nanoparticles-caused elevated H_2_O_2_ by enhancing the activity of enzyme involved in superoxide-dismutation (SOD), H_2_O_2_-metabolizing enzymes (catalase, CAT; and APX), increasing GSH-regeneration (as a result of increased GSH reductase activity; and consequently decreasing GSH-oxidation), finally inducing the transcripts of *Cu/Zn SOD, GSH1, CAT1*, and *GR1* (Li et al.). In a comprehensive *in silico* study, APX and GSH-peroxidase (GPX) genes/proteins from 18 different plant species were identified and compared in order to unravel their significance in excessive H_2_O_2_ management (Ozyigit et al.). Notably, APX and GPX were found to be involved in the metabolism of antioxidants and secondary metabolites, redox homeostasis, stress adaptation, and photosynthesis/respiration. The major redox proteins namely plant peroxiredoxins (Prxs) and sulfiredoxins (Srxs) are involved in antioxidant defense and redox signaling in stressed plants. Srxs were are also found to be involved in antioxidant defense and redox signaling in response to environmental stimuli; post-translational modifications of Srxs regulate the ROS-transduction and bioactivity. On the other hand, Prxs are sensitive to glutathionylation. Investigation of the glutathionylation of recombinant chloroplastic 2-Cys Prx and mitochondrial Prx IIF of pea plants revealed glutathionylation-mediated change of the decameric form of 2-Cys Prx into its dimeric glutathionylated form. Additionally, the reduced dimeric form of Prx IIF was glutathionylated without changing its oligomeric state (Calderón et al.). Thus, glutathionylation was argued to depend on the GSH/GSSG ratio owing to the perceptible difference in the exact effect on the 2-Cys Prx and Prx IIF proteins.

H_2_O_2_-priming (exposure of seeds, seedlings, or plants to stressors/chemical compounds that makes them ready to tolerate the later stress events) helps in biotic and abiotic stress tolerance in various crop plants by triggering the ROS scavenging machinery (Dikilitas et al., 2020). Exogenous supply of H_2_O_2_ can induce stress tolerance under salt, drought, chilling, high temperatures, and heavy metal stress (Hossain et al.). In a study on mustard (*Brassica juncea* L.) cultivars, H_2_O_2_-induced reversal of the major negative impacts of Ni stress (200 mg Ni kg^−1^ soil) led to increased photosynthetic nitrogen-use efficiency, sulfur-use efficiency, and GSH content and decreased levels of lipid peroxidation and electrolyte leakage (Khan et al.). Notably, H_2_O_2_ priming-mediated increased tolerance to cadmium-caused oxidative stress in *Brassica napus* involved fine-tuning between the glyoxalase system and the components of ROS-scavenging machinery (Hasanuzzaman et al.).

## H_2_O_2_ Crosstalk With Other Molecules

Along with H_2_O_2_, other signaling molecules (such as nitric oxide, NO; and calcium, Ca^2+^) and phytohormones (such as jasmonic acid, JA; salicylic acid, SA; and abscisic acid, ABA) play key roles in stress signaling cascades and crosstalk during plants' stress responses (Saxena et al., 2016). To this end, the crosstalk of H_2_O_2_ with NO and Ca^2+^ was argued to contribute to regulation of the plant development and abiotic stress responses (Niu and Liao). Notably, the role of SA in adventitious root formation involved H_2_O_2_ acting as a downstream messenger (Yang et al., 2013). Having emerged as a master regulator of stress responses, ABA signaling pathway triggers significant changes in gene expression and plants' adaptive physiological responses (Saxena et al., 2016). There occurs a close relation among the MAPK cascades, ABA, JA, SA, and H_2_O_2_ where exogenous application of H_2_O_2_ triggers MAPK cascade, which in turn involves ABA, JA, and SA (Saxena et al.). ABA-induced H_2_O_2_ accumulation can protect plant parts (such as pumpkin-grafted cucumber leaves) against Ca(NO_3_)_2_ via ABA/H_2_O_2_ signaling-led induction of ROS-scavenging machinery (Shu et al.). *S*-nitrosoglutathione reductase (GSNOR) determines the level of *S*-nitrosothiol and thereby regulates NO-signaling in plants (Lindermayr, 2018; Jahnová et al., 2019). In *A*. *thaliana*, H_2_O_2_ *in vitro* led to inhibition of the activity of GSNOR and significantly changed NO-homeostasis, which in turn resulted in the activation of ROS-scavenging machinery in order to suppress the oxidative damage (Kovacs et al.).

## Conclusions and Future Perspective

In the current Research Topic “*Recent insights into the double role of hydrogen peroxide in plants*,” the contributions discussed the versatile role of H_2_O_2_ as a signaling molecule that triggers the upregulation of the components of antioxidant defense machinery and imparts tolerance in crop plants against the variety of environmental cues. The crosstalk of H_2_O_2_ with other signaling molecules and phytohormones leads to signal transduction in response to various stresses and regulates plant growth, development, and stress tolerance. Therefore, further understanding on the coordination of H_2_O_2_ and other signaling molecules NO, ${\text{Ca}}_{,}^{2+}$ MAPK, SA, and ABA can pave the way to achieving tolerance in crop plants to increasing stress conditions.

## Author Contributions

NA and SG prepared the first draft of the manuscript. FC, CO-V, LH, NT, AS, MH, and MF read and revised the manuscript. All authors listed approved the final version for publication.

## Conflict of Interest

The authors declare that the research was conducted in the absence of any commercial or financial relationships that could be construed as a potential conflict of interest.

## Publisher's Note

All claims expressed in this article are solely those of the authors and do not necessarily represent those of their affiliated organizations, or those of the publisher, the editors and the reviewers. Any product that may be evaluated in this article, or claim that may be made by its manufacturer, is not guaranteed or endorsed by the publisher.

## References

[B1] Arasimowicz-Jelonek M. Floryszak-Wieczorek J. Deckert J. Rucińska-Sobkowiak R. Gzyl J. Pawlak-Sprada S.. (2012). Nitric oxide implication in cadmium-induced programmed cell death in roots and signaling response of yellow lupine plants. Plant Physiol. Biochem. 58, 124–134. 10.1016/j.plaphy.2012.06.018 22819859

[B2] Baliardini C. Meyer C.-L. Salis P. Saumitou-Laprade P. Verbruggen N. (2015). CATION EXCHANGER1 cosegregates with cadmium tolerance in the metal hyperaccumulator Arabidopsis halleri and plays a role in limiting oxidative stress in Arabidopsis spp. Plant Physiol. 169, 549–559. 10.1104/pp.15.01037 26162428 PMC4577435

[B3] Bienert G. P. Møller A. L. Kristiansen K. A. Schulz A. Møller I. M. Schjoerring J. K.. (2007). Specific aquaporins facilitate the diffusion of hydrogen peroxide across membranes. J. Biol. Chem. 282, 1183–1192. 10.1074/jbc.M603761200 17105724

[B4] Dikilitas M. Simsek E. Roychoudhury A. (2020). “Modulation of abiotic stress tolerance through hydrogen peroxide,” in Protective Chemical Agents in the Amelioration of Plant Abiotic Stress: Biochemical and Molecular Perspectives, eds. A. Roychoudhury and D. K. Tripathi (New York, NY: John Wiley & Sons), 147–173. 10.1002/9781119552154.ch7

[B5] Jahnová J. Luhová L. Petrivalský M. (2019). S-nitrosoglutathione reductase - the master regulator of protein S-nitrosation in plant NO-signaling. Plants 8:48. 10.3390/plants8020048 30795534 PMC6409631

[B6] Keunen E. Remans T. Opdenakker K. Jozefczak M. Gielen H. Guisez Y.. (2013). A mutant of the Arabidopsis thaliana LIPOXYGENASE1 gene shows altered signalling and oxidative stress related responses after cadmium exposure. Plant Physiol. Biochem. 63, 272–280. 10.1016/j.plaphy.2012.12.005 23314084

[B7] Lindermayr C (2018). Crosstalk between reactive oxygen species and nitric oxide in plants: key role of S-nitrosoglutathione reductase. Free Radic. Biol. Med. 122, 110–115. 10.1016/j.freeradbiomed.2017.11.027 29203326

[B8] Miller G. Shulaev V. Mittler R. (2008). Reactive oxygen signaling and abiotic stress. Physiol. Plant. 133, 481–489. 10.1111/j.1399-3054.2008.01090.x 18346071

[B9] Neill S. Desikan R. Hancock J. (2002). Hydrogen peroxide signalling. Curr. Opin. Plant Biol. 5, 388–395. 10.1016/S1369-5266(02)00282-0 12183176

[B10] Opdenakker K. Remans T. Vangronsveld J. Cuypers A. (2012). Mitogen-activated protein (MAP) kinases in plant metal stress: regulation and responses in comparison to other biotic and abiotic stresses. Int. J. Mol. Sci. 13, 7828–7853. 10.3390/ijms13067828 22837729 PMC3397561

[B11] Saxena I. Srikanth S. Chen Z. (2016). Cross talk between H_2_O_2_ and interacting signal molecules under plant stress response. Front. Plant Sci. 7:570. 10.3389/fpls.2016.00570 27200043 PMC4848386

[B12] Sehar Z. Jahan B. Masood A. Anjum N. A. Khan N. A. (2021). Hydrogen peroxide potentiates defense system in presence of sulfur to protect chloroplast damage and photosynthesis of wheat under drought stress. Physiol. Plant 172, 922–934. 10.1111/ppl.13225 32997365

[B13] Sharma P. Jha A. B. Dubey R. S. Pessarakli M. (2012). Reactive oxygen species, oxidative damage, and antioxidative defense mechanism in plants under stressful conditions. J. Bot. 10.1155/2012/217037

[B14] Tamás L. Dudíková J. Durceková K. Halusková L. Huttová J. Mistrík I. (2009). Effect of cadmium and temperature on the lipoxygenase activity in barley root tip. Protoplasma 235, 17–25. 10.1007/s00709-008-0027-2 19067105

[B15] Yang W. Zhu C. Ma X. Li G. Gan L. Ng D.. (2013). Hydrogen peroxide is a second messenger in the salicylic acid-triggered adventitious rooting process in mung bean seedlings. PLoS ONE 8:e84580. 10.1371/journal.pone.0084580 24386397 PMC3874037

